# The ethical considerations of integrating artificial intelligence into surgery: a review

**DOI:** 10.1093/icvts/ivae192

**Published:** 2025-02-25

**Authors:** Arian Arjomandi Rad, Robert Vardanyan, Thanos Athanasiou, Jos Maessen, Peyman Sardari Nia

**Affiliations:** Department of Translational Health Sciences, University of Bristol, Bristol, UK; Department of Surgery and Cancer, Imperial College London, London, UK; Department of Cardiothoracic Surgery, Maastricht University Medical Center, Maastricht, Netherlands; Research Unit, Heart Team Academy, Maastricht, Netherlands; Department of Surgery and Cancer, Imperial College London, London, UK; Department of Surgery and Cancer, Imperial College London, London, UK; Department of Cardiothoracic Surgery, Maastricht University Medical Center, Maastricht, Netherlands; Research Unit, Heart Team Academy, Maastricht, Netherlands; Department of Cardiothoracic Surgery, Maastricht University Medical Center, Maastricht, Netherlands; Research Unit, Heart Team Academy, Maastricht, Netherlands

**Keywords:** Artificial Intelligence, Machine Learning, Medical Ethics, Surgery

## Abstract

The integration of artificial intelligence (AI) into surgery raises significant ethical concerns, including the impact on autonomy, human authority and the patient–doctor relationship. This study underscores the need for a multidisciplinary approach to navigate these ethical dilemmas, involving stakeholders from various fields. A comprehensive literature review up to March 2024 was conducted to assess the ethical implications of AI applications in surgery. This included an examination of data privacy, informed consent, algorithmic bias, the role of advanced robotics, and the impact on surgeons’ decision-making. The study also considered the development of autonomous surgical robots and their ethical implications. The review highlights that while AI can enhance surgical precision and improve clinical decision-making, it also poses several ethical challenges. AI’s ability to support decision-making risks undermining surgeons’ autonomy and judgement, raising concerns about over-reliance on technology. Issues such as data privacy, algorithmic bias and equitable access to AI-driven tools were identified as key ethical concerns. Autonomous surgical robots, while promising, introduce complex questions about accountability and liability, particularly when unexpected outcomes occur. Effective integration of AI into surgical practices demands the development of ethical frameworks that respect both the capabilities of AI and the irreplaceable value of human judgement. Balancing technological advancement with ethical integrity is essential to safeguard patient-centred care and ensure equitable access to AI benefits in healthcare.

## Graphical Abstract

**Figure:**
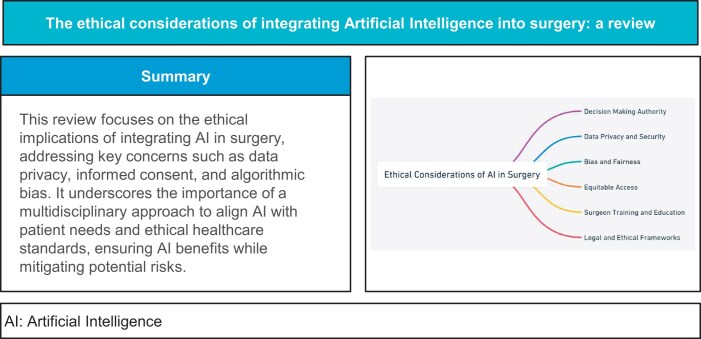

## INTRODUCTION

The advent of artificial intelligence (AI) in the field of surgery represents a transformative shift poised to augment the capabilities of surgeons through enhanced analytical support. This includes aiding in risk assessment, forecasting patient outcomes and providing direct assistance during surgical procedures [1–5]. Such advancements, while promising, introduce substantial ethical complexities. Unlike humans, AI systems do not possess the ability to morally reflect upon experiences and exercise ethical judgement, presenting a unique set of ethical challenges [6, 7]. These challenges are not merely theoretical but have practical implications akin to the ethical dilemmas faced by autonomous vehicles in no-win scenarios.

Human decision-making in medicine is not solely based on factual knowledge; it incorporates a myriad of factors, including emotions, cultural contexts, moral beliefs and personal experiences. This human-centric approach is fundamental to medical ethics. In contrast, AI operates based on algorithms and data, lacking these inherently human elements [8].

The central ethical question then becomes whether AI should be entrusted with decision-making roles, especially considering the trade-off between AI’s precision and efficiency and the potential loss of human touch in ethical considerations. The current objective is to develop an ethical framework where AI complements and enhances human decision-making in surgery without infringing upon the ethical principles that are deeply embedded in human judgement and experience. This entails a careful balance ensuring that AI does not autonomously make decisions that should be grounded in human ethics.

As we progress, the integration of AI in surgical practice is not just a matter of technological evolution but also involves redefining the ethical landscape of medical practice to ensure alignment with fundamental human values [8].

## METHODS

This narrative review explores the ethical considerations of AI in surgery, with an emphasis on cardiothoracic surgery. A non-systematic approach was adopted to identify relevant literature. Articles were sourced from Medline, EMBASE and Google Scholar, covering publications up to March 2024. Search terms included ‘AI in surgery’, ‘ethical implications’, ‘machine learning’, ‘data privacy’ and ‘autonomous surgical robots’. This search yielded 42 relevant articles, which were subsequently screened for relevance based on their titles and abstracts.

The full text of the selected articles was reviewed to extract key ethical themes related to AI. Recurrent themes, including data privacy, informed consent, algorithmic bias and the influence of AI on clinical decision-making, were identified through thematic analysis. These themes were synthesized into a cohesive framework to highlight the core ethical challenges posed by AI in surgical practice.

Both manual review and AI-assisted methods were employed in the review process. ChatGPT, an AI language model, was used to assist in drafting and structuring the manuscript based on the identified data. The authors performed the final review and refinement of the content to ensure the accuracy and integrity of the conclusions drawn.

### Ethical considerations: basic implementations of artificial intelligence in surgery

#### Data analysis and performance metrics

AI’s primary contributions to surgery have emerged through data analysis and performance evaluation. In cardiothoracic surgery, AI’s ability to analyse vast datasets offers insights into surgical outcomes, facilitating the early prediction of complications and supporting clinical decisions, as highlighted by Kishor and Chakraborty [9].

However, the use of AI to evaluate surgeons’ performance introduces ethical concerns. For instance, AI might prioritize efficiency metrics such as operative times over techniques that, while longer, provide better long-term patient outcomes. This raises questions about whether AI-driven metrics could unduly influence surgical decision-making, potentially compromising patient care in favour of efficiency, as discussed by Pedrett *et al.* [10].

Furthermore, the use of AI-generated data by external entities, such as hospital management or insurers, could lead to biased evaluations of surgeons. This may pressure surgeons to alter their practices to meet AI-driven metrics, rather than focusing on patient-centred care, which threatens patient autonomy and holistic clinical judgement.

Finally, the effectiveness of AI depends on the quality and accuracy of the data on which it is trained on. Cardiothoracic surgery databases, such as the Society of Thoracic Surgeons (STS) Database and EACTS Adult Cardiac Database, must ensure that AI systems are fed high-quality, representative data to avoid the perpetuation of biases or inaccuracies. Collaborative efforts to create standardized and validated datasets across institutions will be key to realizing AI’s full potential in cardiothoracic surgery.

### Ethical considerations: intermediate-level artificial intelligence applications in surgery

#### Surgical planning and decision support

As AI advances to an intermediate level within the surgical sphere, its function transitions from mere data analysis to a more dynamic role in surgical planning and decision-making. AI systems are now equipped to furnish surgeons with real-time data, predictive analytics and decision-support mechanisms throughout intricate surgical operations. Specifically in the context of cardiothoracic surgery, AI’s application through machine learning (ML) enhances the decision-making process and the precision of surgical interventions as elucidated by Pedrett *et al.* [10].

Recent studies demonstrate AI’s significant role in improving risk stratification using ML models, often outperforming traditional risk scores like EuroSCORE II. For instance, a study comparing ML models such as random forests and elastic nets to EuroSCORE II found that AI-driven models achieved better accuracy in predicting operative mortality and complications, such as acute kidney injury, in cardiac surgery patients [11, 12].

AI also facilitates the creation of new prediction models that incorporate a wider array of patient data, improving accuracy and outcomes. Another study systematically reviewed AI’s application in cardiac surgeries, highlighting that AI not only enhances outcome predictions but also aids in optimizing surgical decisions based on patient-specific data [13].

Moreover, AI-driven decision-making in the heart team ensures that all available data and evidence are considered, resulting in more precise and effective treatment plans. An example includes the prediction of perioperative myocardial injury using ML algorithms, which enabled surgeons to adjust strategies to mitigate risk in real-time [14].

However, this amalgamation of AI and surgical practice is not devoid of ethical considerations. An illustrative ethical dilemma emerges when an AI system proposes an unconventional surgical strategy, derived from its analysis of analogous historical cases. Conversely, the surgeon, relying on extensive experience and personal evaluation of the patient, may hold a divergent viewpoint. This discrepancy poses an ethical quandary: should the surgeon adhere to the AI-generated recommendation, predicated on collective data, or depend on their own clinical acumen? The burden of the ultimate decision and its ramifications represents a profound ethical challenge, accentuating the imperative for explicit protocols governing AI’s influence on decision-making processes. Hashimoto *et al.* [15] acknowledge AI’s potential to transform surgical practices by augmenting decision-making capabilities, yet they also stress the necessity for surgeons to appraise AI contributions critically.

#### Patient data management and the success of artificial intelligence depends on data sharing

Within the domain of intermediate-level AI applications, a significant emphasis is placed on the sophisticated management and sharing of patient data to facilitate tailored surgical strategies. AI systems not only assist in intraoperative decision-making but also play a crucial role in postoperative management, particularly in the ICU. Here, they analyse vast amounts of data generated per second from patient monitoring, predicting deterioration before it happens and allowing for timely interventions. For example, ML models have been applied in ICUs to predict extubation failure, monitor mechanical ventilation and forecast neurological outcomes, providing real-time support in managing cardiothoracic surgery patients [16, 17]. These AI tools enhance decision-making by processing patient data in real-time, improving outcomes and minimizing complications in critical care environments [16, 18].

This advancement foregrounds pivotal concerns regarding data privacy and security. In cardiovascular medicine, the ethical and legal quandaries associated with patient privacy and security, particularly in the context of big data, are underscored by Tat and Rabbat [19]. The ethical stewardship of such data is of utmost importance, as any compromise or misapplication could culminate in grave privacy infringements and erode patient confidence.

Furthermore, the successful implementation of AI in healthcare, including cardiothoracic surgery, hinges on the availability and sharing of data. However, this is challenging due to regulatory constraints such as the General Data Protection Regulation (GDPR) in the European Union. One approach to overcoming these challenges is through the development of secure data-sharing frameworks that comply with regulatory standards. These frameworks ensure that data are shared in a way that protects patient privacy and adheres to legal requirements. Fostering international collaborations and creating anonymized datasets can help facilitate data sharing while protecting patient privacy. This collaborative approach can drive the advancement of AI technologies and their integration into clinical practice, ultimately improving patient outcomes.

The question of informed consent is also paramount. It is essential that patients are comprehensively briefed on the utilization of their data by AI systems within their surgical treatment. This disclosure must be conveyed in a manner that is both accessible and clear, ensuring that patients are truly informed and can extend meaningful consent. This is particularly salient in medical imaging, where Tripathi and Musiolik [20] illuminate ethical dilemmas stemming from algorithmic biases.

An additional ethical concern is the potential for intrinsic biases within AI algorithms. The impartiality of AI systems is contingent upon the neutrality of the training data. Should these data embody historical prejudices, such biases may be replicated in surgical advisories. In healthcare scenarios, AI recommendations predicated on data that fails to accurately reflect the patient’s demographic could result in suboptimal or detrimental surgical planning, a scenario explored by Naik *et al.* [21]. The imperative to ensure fairness and address biases in AI algorithms is critical for the provision of equitable healthcare. These challenges underscore the necessity for a synergistic interplay between technological advancements and human expertise, highlighting the critical role of human oversight and ethical integrity in the rapidly advancing arena of surgical AI.

### Ethical considerations in advanced artificial intelligence applications in surgery

#### Autonomous or semi-autonomous surgical robots

At the forefront of AI in surgery, future advanced applications manifest through autonomous or semi-autonomous surgical robots, significantly minimizing the need for human intervention. Shademan *et al.* [22] have exemplified the prowess of such systems with the autonomous soft-tissue surgical robot, STAR, which has demonstrated superior performance in executing *in vivo* robotic anastomosis with greater precision and consistency than human surgeons. This advancement signals a transformative potential to enhance surgical accuracy and patient outcomes.

However, this innovation introduces complex questions regarding the distribution of responsibility and liability, particularly when an AI system’s independent decision precipitates a negative event. For instance, if a robotic surgery system autonomously modifies a surgical technique during a procedure and this leads to a complication, the delineation of accountability becomes blurred. The determination of liability—whether it falls upon the surgeon, the healthcare institution or the AI developers—necessitates a comprehensive legal and ethical framework to navigate these novel challenges.

Moreover, there is an ethical imperative to ensure that these sophisticated AI systems do not amplify existing disparities within healthcare. The risk that such advanced technology may be accessible only to affluent hospitals in developed regions raises concerns about exacerbating the divide in healthcare quality among different socioeconomic and geographic demographics, underscoring the importance of equitable access to healthcare innovations.

### Surgeon training and education: artificial intelligence-enhanced surgical simulation

The integration of AI into cardiothoracic surgical training is revolutionizing education by offering immersive platforms like Virtual Reality (VR) simulations with ML, enabling trainees to practice complex procedures with real-time feedback. This data-driven approach enhances skill development without requiring constant supervision [23].

However, there are concerns that over-reliance on AI simulations could reduce essential hands-on experience needed for real-world skills. While AI effectively assesses technical proficiency, human judgement and adaptability in complex, patient-specific scenarios remain irreplaceable. A balanced curriculum combining AI-enhanced learning with traditional training is crucial. AI can also standardize education, but it risks overlooking the nuanced decision-making vital for unique cases [24, 25]. Marker-less motion tracking systems, for example, show promise in skill evaluation but are still evolving in cardiothoracic surgery [24–26]. Therefore, AI should complement, not replace, human elements like adaptability and clinical judgement. To maximize AI’s potential, surgeons need comprehensive education on AI technologies, including integrating AI into medical curricula and offering specialized training and continuous professional development.

### Future directions

Addressing the ethical challenges surrounding AI in healthcare requires a multidisciplinary approach. Ethicists ensure that AI tools adhere to the highest moral standards, while clinicians contribute practical insights from patient care, helping to identify potential risks and benefits of AI-based solutions. Data scientists are critical for developing robust, accurate systems, and policymakers guide regulatory frameworks to ensure safety and efficacy. However, patients—key stakeholders in this ecosystem—are often overlooked.

How could we gauge the public’s readiness to start using AI in healthcare? Will there be a generational change, with patients born after the 2000s being more likely to trust AI-driven decision-making as they have lived in a world with AI? Surveys, focus groups and public consultations could be employed to understand patients’ perspectives and readiness to adopt AI in healthcare. Additionally, educational campaigns aimed at raising awareness about the benefits and risks of AI in healthcare can help build trust among the general public.

### Imagination, ethics, and artificial intelligence in surgical decision-making

#### Imagination

Imagination, a distinct human attribute, extends beyond mere creativity to include empathy, ethics and the ability to foresee and navigate moral complexities [9]. This trait has been a cornerstone of ethical decision-making, particularly in surgery, where it melds innovative thinking, empathy, ethical judgement and foresight. Surgeons utilize their imaginative capacities to visualize complex procedures, anticipate complications and empathize with patients, allowing for a nuanced understanding of patient needs and ethical conundrums.

AI introduces a data-driven dimension to surgery, offering enhanced diagnostics, precise analytics and support in intricate surgical tasks. Its promise lies in increasing accuracy and efficiency, potentially reducing human error [1–5]. It could complement human imagination, especially in processing vast data volumes or executing repetitive tasks with precision, thus mitigating error risks.

The prospect of AI possessing a form of imagination is a subject of debate. While AI can identify patterns and predict outcomes, its ‘imagination’ fundamentally differs from the human variety. However, AI’s capacity to generate novel solutions through data analysis and learning algorithms might be viewed as a basic form of imagination. This burgeoning capability might evolve, allowing AI to simulate imaginative processes in problem solving and decision-making.

The comparison between the human mind, shaped by billions of years of evolution and genetic programming, and the potential of AI to achieve a similar level of complexity through advanced programming raises profound questions. It challenges us to ponder whether our minds and brains, sculpted through extensive evolutionary trial and error, are fundamentally dissimilar from a highly complex AI programme potentially capable of mirroring human cognitive processes. This contemplation not only explores the boundaries of AI’s capabilities but also delves into the essence of human cognition and its unique characteristics.

#### Decision-making authority

The integration of AI in surgery raises a critical ethical question: should humans retain decision-making authority, or can it be delegated to AI? This strikes at the core of our need for self-governance, particularly in healthcare. Traditionally, decisions in surgery are made by highly trained surgeons, grounded in empathy and shared human experiences, which form the foundation of the patient–doctor relationship.

AI’s entry into surgery introduces unmatched precision and data analysis capabilities, offering insights beyond human potential [27, 28]. Acting as an intelligent assistant, AI could mitigate human errors caused by emotions or bias. However, ethical decisions in surgery go beyond clinical data, encompassing patient dignity, cultural values, and moral considerations [29, 30]. Surgeons take into account not only medical facts but also the patient’s broader life context, something AI currently cannot fully grasp. Yet, AI’s potential to evolve and engage with ethical reasoning should not be dismissed.

Moreover, AI systems reflect the perspectives and biases of their creators, raising concerns about biased surgical decisions and unequal patient treatment. Darwin’s ‘The Descent of Man’ highlights the importance of morality, shaped by both biology and culture, in human decision-making [31, 32]. Our ethical sense, though influenced by intellectual evolution, is deeply rooted in cultural context. It is conceivable that AI, through its own evolution, could develop a distinct cultural framework, either advancing or undermining moral practice [9].

Thus, AI in surgery is a double-edged sword: a symbol of precision, yet a reminder of the irreplaceable human element. The challenge is to balance AI’s capabilities with the empathetic aspects of patient care, ensuring technology enhances rather than replaces human decision-making. This balance represents the next frontier in AI’s role in surgery.

## CONCLUSIONS

The integration of AI in surgery represents a significant leap in precision and efficiency, offering the potential to revolutionize surgical outcomes. However, it also raises important ethical questions, particularly around data privacy, bias in algorithms and decision-making authority. Balancing AI’s advanced capabilities with the irreplaceable human qualities of empathy, ethical judgement and intuitive decision-making is essential to ensuring that technology enhances, rather than diminishes, the patient experience.

Ethical concerns around patient autonomy, responsibility and informed consent must be carefully managed as AI becomes more prevalent in surgical practices. Additionally, the risk of exacerbating healthcare disparities through algorithmic biases highlights the need for equitable frameworks and vigilant oversight. Central to this discourse is the inclusion of patients, whose perspectives are invaluable in shaping AI technologies that are not only advanced but also responsive to their needs and ethical considerations.

Moving forward, a collaborative effort among ethicists, clinicians, data scientists, policymakers and patients will be essential to navigating the challenges posed by AI. By fostering open dialogue and ensuring transparency in AI’s decision-making processes, we can create a framework where technological progress is aligned with the core values of medicine—preserving human dignity, autonomy and the trust that underpins patient care.

## Data Availability

The data are available from the corresponding author upon request.

## ABBREVIATIONS

AI: Artificial intelligence
GDPR: General Data Protection Regulation
ML: Machine learning
STS: Society of Thoracic Surgeons
